# Deep knowledge tracing with learning curves

**DOI:** 10.3389/fpsyg.2023.1150329

**Published:** 2023-03-30

**Authors:** Hang Su, Xin Liu, Shanghui Yang, Xuesong Lu

**Affiliations:** School of Data Science and Engineering, East China Normal University, Shanghai, China

**Keywords:** knowledge tracing, learning curve theory, three-dimensional convolutional neural networks, capsule networks, deep learning

## Abstract

Knowledge tracing (KT) models students' mastery level of knowledge concepts based on their responses to the questions in the past and predicts the probability that they correctly answer subsequent questions in the future. Recent KT models are mostly developed with deep neural networks and have demonstrated superior performance over traditional approaches. However, they ignore the explicit modeling of the learning curve theory, which generally says that more practices on the same knowledge concept enhance one's mastery level of the concept. Based on this theory, we propose a Convolution-Augmented Knowledge Tracing (CAKT) model and a Capsule-Enhanced CAKT (CECAKT) model to enable learning curve modeling. In particular, when predicting a student's response to the next question associated with a specific knowledge concept, CAKT uses a module built with three-dimensional convolutional neural networks to learn the student's recent experience on that concept, and CECAKT improves CAKT by replacing the global average pooling layer with capsule networks to prevent information loss. Moreover, the two models employ LSTM networks to learn the overall knowledge state, which is fused with the feature learned by the convolutional/capsule module. As such, the two models can learn the student's overall knowledge state as well as the knowledge state of the concept in the next question. Experimental results on four real-life datasets show that CAKT and CECAKT both achieve better performance compared to existing deep KT models.

## 1. Introduction

Knowledge tracing (KT) models a student's changing knowledge state *via* tracking her interactions with coursework. By observing a student's answers to a sequence of questions, a KT model can adjust her knowledge state over time and predict her future performance. Due to the ease of interpretation and adoption, the philosophy of knowledge tracing has been widely adopted in intelligent education platforms (Mcgreal et al., 2013; Kaplan and Haenlein, 2016; Zhang Y. et al., 2020).

Knowledge tracing models are used to be constructed using statistical cognitive modeling methods such as Bayesian inference with a Hidden Markov model (Corbett and Anderson, 1994; Yudelson et al., 2013) and factor analysis using logistic regressions (Cen et al., 2006; Pavlik et al., 2009; Chi et al., 2011). In the past few years, researchers have turned to train neural networks for knowledge tracing due to the availability of massive educational data released by large MOOC platforms and educational institutions. In the pioneering work (Piech et al., 2015), Piech et al. propose the DKT model using an LSTM network, which significantly improves the overall accuracy of predicting students' responses to questions. Inspired by this work, a series of deep learning models have been proposed to target various aspects in the knowledge tracing task, including DKVMN (Zhang et al., 2017), EKT (Huang et al., 2019), AKT (Ghosh et al., 2020), CKT (Shen et al., 2020), IEKT (Long et al., 2021), and CL4KT (Lee et al., 2022), etc.

### 1.1. The motivation

Despite the major advances in predicting student performance, we notice that existing deep KT models ignore the explicit modeling of the “learning curve” theory, which generally states that more practices would bring more improvement on a skill. Such improvement may manifest itself as taking less and less time to complete tasks that require the same skill, or resulting in a smaller and smaller error rate, etc. Newell and Rosenbloom (Newell and Rosenbloom, 1981) first theorize this ubiquitous phenomenon and find that the error rate of performance and the amount of practices have a power relationship in diverse learning tasks. They depict the relationship using the following simple equation:


(1) $$\begin{array}{l} Y=aX^{-b}, \end{array}$$

Where *Y* denotes the error rate or the cost to complete a task, *X* is the number of previous trials using the skill needed by the task, *a* is the difficulty of the skill and *b* is the learning rate of the skill. Similarly, the learning curve phenomenon may also be depicted using other equations such as exponential rise or fall and Sigmoid curve (Ritter and Schooler, 2002; Leibowitz et al., 2010) based on different assumptions. Nevertheless, they all reveal that the more one practices on a skill, the better she performs on it. According to the theory, in the scenario of education, we may expect that the more questions a student answers about the same knowledge concept, the less likely she will make mistakes on the concept, i.e., the mastery of the knowledge concept is enhanced.

**Figure 1:**
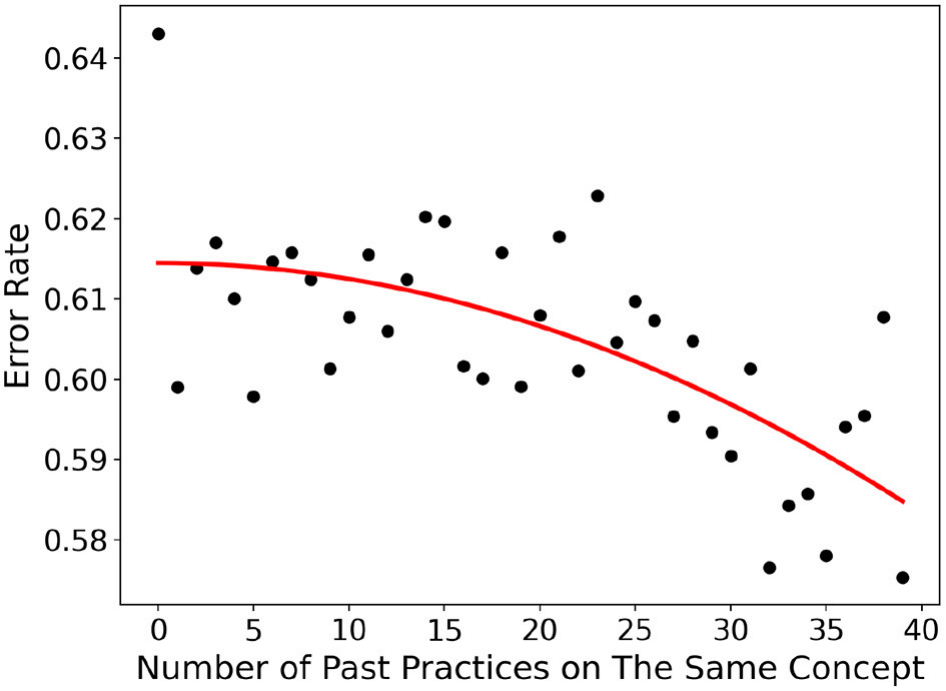
The “learning curve” in ASSISTments2017.

Figure 1 shows a “learning curve” in the ASSISTments2017 dataset. The x-axis is the number of past practices on the same concept, and the y-axis is the averaged error rate when a student responds to the next question associated with the concept. It can be observed that the error rate decreases generally with the increased number of past practices, and the fitted curve clearly shows the learning curve phenomenon.

Inspired by this phenomenon, we hypothesize that if we incorporate the feature of past practices on the same knowledge concept into knowledge tracing, we can better model such enhanced mastery of the concept, thereby promoting the overall accuracy for estimating the mastery levels of knowledge concepts. Therefore, we propose to model the learning curve theory to augment knowledge tracking, i.e., learning a student's past experience on the concept of the next question to be answered. Surprisingly, we find that this simple idea has not been sufficiently explored in existing models. DKT (Piech et al., 2015) and DKVMN (Zhang et al., 2017) have not given a particular bias to a student's past experience of applying the knowledge concept in the next question. In the models such as EERNN (Su et al., 2018), EKT (Huang et al., 2019), SAKT (Pandey and Karypis, 2019), and AKT (Ghosh et al., 2020), the heterogeneous impacts of the past interactions are modeled using the attention mechanism. As such, the prediction to the next question may be influenced by the past practices on the questions with a different concept. SKVMN (Abdelrahman and Wang, 2019) and DKT+forgetting (Nagatani et al., 2019) have attempted to explicitly model the impact of past performance on the same knowledge concept. However, SKVMN needs to empirically define a threshold for the similarity between two concepts and then pick the past similar trials based on the threshold, whereas DKT+forgetting uses handcrafted features to represent repeated practices on the same concept. Both of them rely on empirically-determined settings.

### 1.2. Our solution

To bridge this gap, we propose to explicitly model a student's experience on the same knowledge concept with the one covered by the next question, and combine the experience with the student's overall knowledge state to predict her response to the next question. To learn the overall knowledge state, without loss of generality, we train a two-layer LSTM network, where the input at each step is the embedding of a student-question interaction. To learn the knowledge state pertaining to a particular concept, we need another module to extract the feature from previous practices on the concept. One may use RNNs/LSTM (Rumelhart et al., 1986; Hochreiter and Schmidhuber, 1997), self-attention networks (Vaswani et al., 2017), or CNNs (LeCun et al., 1989) to capture the feature. However, when using RNNs/LSTM or self-attention, the input embeddings of student-question interactions are the same with that of the two-layer LSTM network, so that the extracted latent features are likely to be homogeneous and bring limited performance gain. When using CNNs, a common method is to arrange the input embeddings at all steps into a matrix (feature map) and apply convolution operations on it. In this way, neither the knowledge state contained in each embedding nor the evolution of the knowledge state is well captured because the convolution filters only focus on the local patterns in the feature map.

To enable better modeling of the knowledge state pertaining to the same concept, we design two specific modules and form two models. In the first model, inspired by the video processing tasks (Tran et al., 2015, 2018), which extract features from a sequence of frames, we propose to use three-dimensional convolutional neural networks (3D-ConvNets) for capturing the information from the recent student-question interactions on the same concept. Specifically, when predicting a student's response to the next question at time *t*+1 pertaining to a knowledge concept *c*, we fetch *k* recent interactions of her before *t*+1 that also cover *c*, and represent them using vectorized embeddings. Then we reshape the embeddings into matrices and stack them in the chronological order to form a three-dimensional tensor. This tensor represents the student's experience on applying *c* in the past. We then adopt a 3D-ConvNets module to extract the latent knowledge state of the student on the concept *c* from the tensor and use a standard global average pooling layer to obtain the two-dimensional knowledge state.

The rationale behind the design of the 3D-ConvNets module can be seen as follows. On the one hand, we reshape the embeddings into matrices so that we could leverage the convolutional module to extract heterogeneous features from those extracted by the LSTM network. On the other hand, the 3D-ConvNets use the first two dimensions of the filters to capture the latent knowledge state in each reshaped matrix (i.e., each step) and use the third dimension to capture the evolution of the knowledge state. As such, the 3D-ConvNets module may overcome the aforementioned problems of using RNNs/LSTM, self-attention networks, or CNNs.

The global average pooling layer after the 3D-ConvNets module in the first model may introduce information loss on the knowledge state tensor. As such, in the second model, we further propose to replace the pooling layer with capsule neural networks (CapsNets) and route dynamically the feature maps produced by the 3D-ConvNets module. Specifically, the output tensors of the 3D-ConvNets module are viewed as multiple types of capsules, where the same type of capsules form a two-dimensional grid. Then we devise a CapsNets module consisting of a convolution layer and a voting (dynamic routing) layer to transform the tensors into the two-dimensional knowledge state. As such, rather than using pooling, each element in the final knowledge state is generated *via* voting among the elements at the same position of different feature maps, thereby circumventing the information loss problem induced by the pooling layer.

Using the above two methods, we explicitly model the learning curve theory by only extracting the feature from past trials on *c*. Denote this latent feature by **m**_*t*_ and denote by **h**_*t*_ the overall knowledge state output by the first LSTM layer, we use a fusion gate to fuse **m**_*t*_ and **h**_*t*_, which decides how much information of each feature is fed into the second LSTM layer. The intuition is that we need to combine both the overall knowledge state since there might be other concepts relevant to *c*, and the particular knowledge state of *c* since the past experience of applying *c* largely affects the next response. Finally, the output of the second LSTM layer is transformed to predict the student's response to the next question at time *t*+1.

For the convenience of presentation, below we refer to the first model using only the 3D-ConvNets module as *Convolution-Augmented Knowledge Tracing*, or simply CAKT, and refer to the second model using both the 3D-ConvNets module and the CapsNets module as *Capsule-Enhanced Convolution-Augmented Knowledge Tracing*, or simply CECAKT.

In the experimental section, we demonstrate that both CECAKT and CAKT outperform main existing deep KT models as well as their alternative architecture designs. Between the two models, CECAKT improves CAKT on all the experimental datasets. Particularly, when students have a lot of repeated practices on the same knowledge concept, the two models perform much better than the comparative models, which confirms the effectiveness of explicitly modeling the learning curve theory.

### 1.3. Summary of contribution

Our contribution in this work can be briefly summarized as follows.

1. We reveal the “learning curve” phenomenon in the real-life dataset, which motivates us to propose the explicit modeling of the learning curve theory in the deep knowledge tracing model. In particular, when predicting a student's response to a question pertaining to a knowledge concept, we collectively learn the latent feature of the student's historical performance on the same concept and the latent feature of her overall knowledge state (see Section 1.1).
2. We propose two novel models, namely CAKT (see Section 4) and CECAKT (see Section 5), that use three-dimensional convolutional neural networks and capsule networks to learn from the reshaped embeddings of the student-question interactions regarding the same knowledge concept. To the best of our knowledge, we are the first to introduce such an architecture in the KT task. We discuss the rationale behind the particular architecture designs (see Sections 1.2, 4, and 5).
3. We conduct the experiments to show the two models have the overall best performance compared with the existing deep KT models, and CECAKT improves CAKT on all the experimental datasets. We analyze the datasets and discuss when the two models can perform particularly better and when the performance gain may be limited (see Section 6.5.1). The results further confirm the effectiveness of explicit modeling the learning curve theory. We also conduct an ablation study to justify the choice of model components (see Section 6.7) and illustrate the impact of modeling the learning curves *via* use cases (see Section 6.8).

## 2. Related work

### 2.1. Deep learning for knowledge tracing

The Deep Knowledge Tracing (DKT) model (Piech et al., 2015) first applies deep learning on the task. DKT uses an LSTM network (Graves et al., 2013; Sutskever et al., 2014) to learn from the student-question interaction sequences. At each step, the LSTM unit takes as input an interaction tuple representing which question is answered and whether the answer is correct. The output is a vector of length equal to the number of knowledge concepts, where each element is a probability representing the predicted mastery level of a concept. When predicting a student's response to the next question, the element of the output vector corresponding to the concept covered by the question is used to predict the probability of correctness.

Then inspired by memory-augmented neural networks (Graves et al., 2016; Santoro et al., 2016; Zhang et al., 2017) propose Dynamic Key-Value Memory Networks (DKVMN) to improve DKT's structure. DKVMN uses two memory matrices, where one is static and used to store the latent knowledge concepts of the questions, and the other is dynamic and used to represent the student knowledge state. DKVMN updates the knowledge state of a student by reading from and writing to the dynamic matrix. Following this work, Abdelrahman and Wang (2019) propose Sequential Key-Value Memory Networks (SKVMN) to capture the dependencies between questions. They assume that the predicted response to the next question only depends on the previous interactions to the questions with similar concepts. At each step, they introduce an additional hop-LSTM network before the output layer of DKVMN, whose LSTM units connect only the hidden states of the steps pertaining to the similar questions.

More studies investigate the utility of recently proposed architectures. For example, Pandey and Karypis (2019) propose Self-Attentive Knowledge Tracing (SAKT), with the hope to handle the data sparsity problem by using the Transformer architecture (Vaswani et al., 2017). Ghosh et al. (2020) propose Attentive Knowledge Tracing (AKT), which uses a series of attention networks to draw connections between the next question and past interactions. Nakagawa et al. (2019) propose Graph-based Knowledge Tracing (GKT). They construct a graph to connect related concepts. When a student answers a question associated with a particular concept, GKT updates simultaneously the student's knowledge state of the concept and the related concepts. Guo et al. (2021) propose Adversarial Training based Knowledge Tracing (ATKT) to avoid the risk of overfitting in existing deep KT models. They construct adversarial examples by adversarial perturbations, and use them with the original examples to jointly train ATKT. To tackle the problem of sparse interactions between students and questions, Lee et al. (2022) propose several learning history augmentation methods and introduce a contrastive learning framework for knowledge tracing. The proposed CL4KT model reveals semantically similar or dissimilar examples of a learning history and stimulates to learn their relationships.

Another line of work attempts to incorporate additional features into the input. For example, EERNN (Su et al., 2018) uses a Bi-LSTM network to obtain the text embedding of each question, and concatenates the embedding with that of the corresponding student-question interaction. EKT (Huang et al., 2019) extends the idea and replaces the hidden states in the LSTM network with hidden matrices. The DKT+forgetting (Nagatani et al., 2019) model uses manually-constructed features pertaining to the forgetting behavior in the learning process, and feeds the features as additional information into the DKT model. MsaCNN (Zhang et al., 2023) captures structured features from student grade records and discovers relationships between courses, and finally integrates multi-source features to predict learning performance. Other work of this line includes DKT-DSC (Minn et al., 2018), CKT (Shen et al., 2020), and HGKT (Tong et al., 2022), etc.

More recent studies focus more on simulating the learning theories and behaviors. Wang et al. (2021) propose the HawkesKT model, which investigates the temporal cross-effects between different knowledge concepts, inspired by the fact that each past interaction has a different time-sensitive impact on the next interaction. Long et al. (2021) propose Individual Estimation Knowledge Tracing (IEKT), which estimates a student's knowledge state before answering a question and assesses her sensitivity to knowledge acquisition before updating the knowledge state. Shen et al. (2021) propose Learning Process-consistent Knowledge Tracing (LPKT), which monitors the knowledge state by directly simulating the learning process and provides a new paradigm that takes into account the consistency of students' changing knowledge state. Long et al. (2022) propose Collaborative Knowledge Tracking (CoKT), which makes full use of the student-to-student information for knowledge tracking, i.e., the responses of other students who have answered similar questions.

### 2.2. Three-dimensional convolutional networks

Inspired by the great success of convolutional neural networks in the field of computer vision (Shi et al., 2016; He et al., 2017; Redmon and Farhadi, 2018), three-dimensional convolutional neural networks (Tran et al., 2015, 2018) are proposed to handle video analysis tasks. A video can be viewed as a sequence of images (frames) and formally represented using a D × H × W tensor, where D represents the depth or time of the video, H and W represent the height and width of each frame, respectively. The filters of 3D-ConvNets have also three dimensions accordingly. Tran et al. (2015) demonstrate that 3D-ConvNets can better model the temporal information than two-dimensional convolutional networks. When using two-dimensional convolutions, all the frames are convolved using the same 2D filters and therefore the temporal information is neglected, whereas 3D filters in 3D-ConvNets preserve temporal information during the convolution operations. We therefore leverage this property and use 3D-ConvNets to learn from the sequence of reshaped interaction matrices pertaining to the same knowledge concept, with the expectation that both the knowledge state at each step and the evolution of the knowledge state are learned.

### 2.3. Capsule networks

A capsule is a group of neurons whose vector represents the instantiation parameters of a specific type of entity such as an object or an object part. Based on this property, Sabour et al. (2017) propose a framework of capsule networks that consist of a series of convolutional capsule layers. The capsules in the lower layers encode different partial properties of an object such as pose, texture and deformation, and the capsules in the higher layers aggregate these partial properties and encode more invariant features, such as the existence of a certain object. As such, differently from traditional CNNs, capsule networks are better at encoding the part-whole relationships in the input data. Moreover, the aggregation of capsules in a lower layer into the capsules in a neighbor higher layer is performed using a so-called dynamic routing mechanism, which overcomes the information loss problem induced by the pooling layer in traditional CNNs. Specifically, the output of a capsule in the lower layer is scaled down by coupling coefficients that sum to 1 and gets sent to appropriate capsules in the higher layer accordingly. A larger coupling coefficient means larger contribution that the capsule in the lower layer makes to a capsule in the higher layer. Due to these advantages over traditional CNNs, capsule networks quickly demonstrate superior performance in tasks such as image segmentation (LaLonde and Bagci, 2018; Nguyen et al., 2021), face recognition (Sepas-Moghaddam et al., 2021; Wu et al., 2021; Yu et al., 2022), and sentiment classification (Chen and Qian, 2019; Du et al., 2019; Zhang B. et al., 2020).

## 3. Problem definition

At each step, the knowledge tracing task takes as input a sequence of previous student-question interactions and outputs the prediction of the student's response to the next question. Formally, the problem of knowledge tracing can be defined as follows.

### 3.1. Definition of (deep) knowledge tracing

*For each student, denote by* *q*_*i*_ *the* *i*^*th*^ *question she answers and by* *a*_*i*_ *the corresponding response. At each step* *t, given a sequence of previous student-question interactions* *X* = {*x*_1_, *x*_2_, …, *x*_*t*_}*, where* *x*_*i*_ = (*q*_*i*_, *a*_*i*_)*, knowledge tracing predicts the student's response* *a*_*t*+1_ *to the next question* *q*_*t*+1_*, i.e., the probability* *p*(*a*_*t*+1_ = 1|*q*_*t*+1_, *X*) *that the student correctly answers the next question*.

In the above definition, *a*_*i*_ is a binary variable where 1 represents the student's answer is correct and 0 otherwise, *q*_*i*_ is represented using a one-hot vector **e**_*i*_ with length *M*, where *M* is the number of distinct questions/concepts^1^. Following previous studies (Piech et al., 2015; Zhang et al., 2017; Huang et al., 2019), *x*_*i*_ is encoded using a one-hot vector **x**_*i*_ of length 2*M*. If *a*_*i*_ = 0, we concatenate **e**_*i*_ with a zero vector **z** of length *M* to form **x**_*i*_; otherwise, we concatenate **z** before **e**_*i*_. The encoding process can be summarized as follow:


(2) $$x_{i}=\left\{ \begin{array}{l} {}[e_{i}⊕z]\text{ if }a_{i}=0,\\ {}[z⊕e_{i}]\text{ if }a_{i}=1, \end{array}\right.$$

Where ⊕ represents the concatenation operation.

## 4. The CAKT model

### 4.1. Model overview

**Figure 2:**
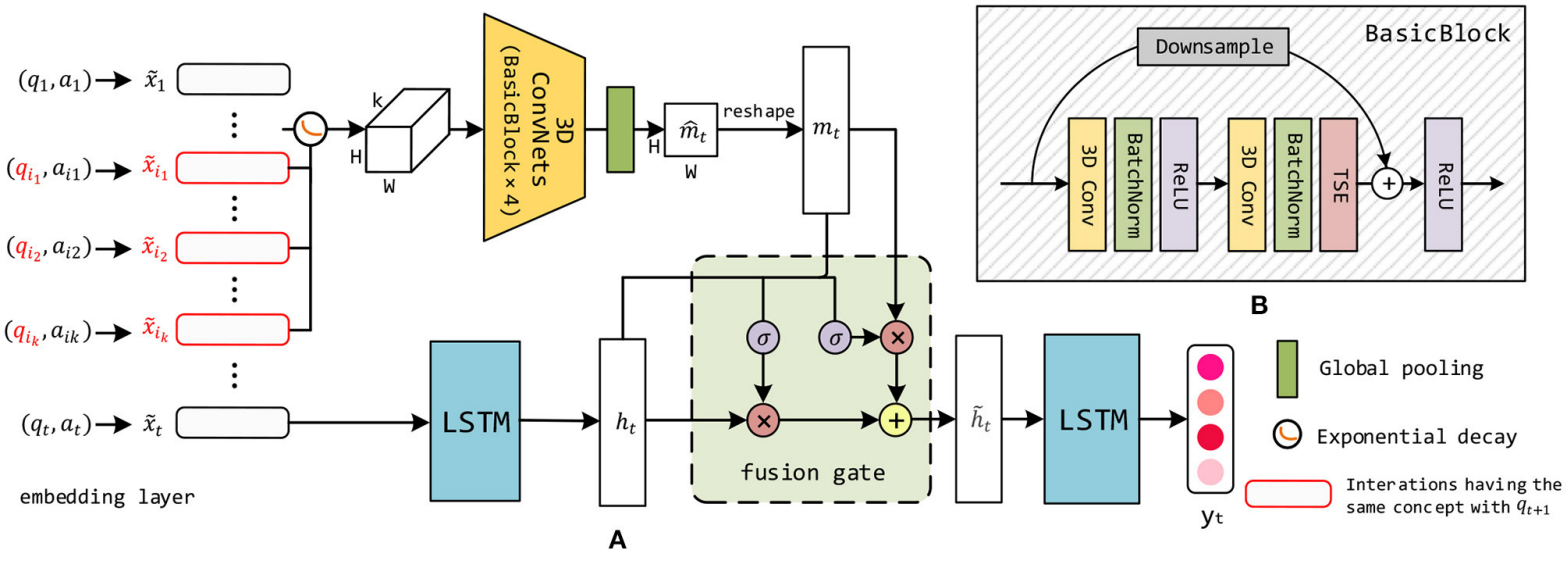
**(A)** The overall architecture of CAKT. The red rounded rectangles represent the embeddings of the interactions that apply the same knowledge concept with that of *q*_*t*+1_. The 3D-ConvNets and the left LSTM layer extract the features m_*t*_ pertaining to the latent knowledge state on the concept covered by *q*_*t*+1_ and the overall latent knowledge state h_*t*_, respectively. The component to the left of the tensor represents the exponential decay function. The 3D-ConvNets module consists of four BasicBlocks depicted in **(B)**. The fusion gate adaptively integrates the two features. Finally, the right LSTM layer transforms the fused feature and outputs the integrated knowledge state y_*t*_. **(B)** Each BasicBlock consists of two 3D convolutional layers, two batch normalization layers, two ReLU layers and one TSE layer. It also employs a residual connection between the input to the block and the output of the TSE layer.

First, we describe the CAKT model (Yang et al., 2022) that adopts a 3D-ConvNets module to model the learning curve, i.e., capturing the feature from past trials on the same knowledge concept. Figure 2A illustrates the architecture of CAKT, which consists of the 3D-ConvNets module (top) and the two-layer LSTM network (bottom). The 3D-ConvNets module functions between the two LSTM layers (left and right LSTM).

In the original input sequence *X* = {**x**_1_, **x**_2_, …, **x**_*t*_}, each interaction **x**_*i*_ is a one-hot vector of length 2*M*, as described in Section 3. We employ an embedding layer to convert each **x**_*i*_ into a dense embedding ${\tilde{\text{x}}}_{i}\in {\mathbb{R}}^{d_{e}}$, as depicted in the leftmost part of the architecture. The reasons are twofold. First, for datasets with a large number of unique knowledge concepts, such as Statics2011 described in Section 6.1, a one-hot encoding can quickly become impractically large (Piech et al., 2015). Second, compared to one-hot encodings, it is much easier for convolutional neural networks to find interesting patterns in the denser representations. Then the first LSTM layer (left LSTM) learns from the entire sequence of embedded interactions $\tilde{X}=\left\{{\tilde{x}}_{1},{\tilde{x}}_{2},\ldots ,{\tilde{x}}_{t}\right\}$, and outputs a latent representation ${\text{h}}_{t}\in {\mathbb{R}}^{d_{h}}$ at each time step *t*, i.e., **h**_*t*_ captures the student's historical performance on all the questions until *t*. This is depicted in the bottom-middle of the architecture. Note that we just draw the LSTM unit at time *t* and omit all previous units for the sake of simplicity.

To incorporate the learning curve theory into the model, when predicting a student's response to *q*_*t*+1_ with knowledge concept *c*, we additionally investigate how she has performed on the *k* most recent questions before time *t*+1 covering the same concept *c*. The structure is depicted in the top part of the architecture. Specifically, we pick the *k* most recent embedded interactions ${\tilde{\text{x}}}_{i_{1}},{\tilde{\text{x}}}_{i_{2}},...,{\tilde{\text{x}}}_{i_{k}}$ that contain *c*, as shown by the red rounded rectangles in the left part of the architecture. If there are less than *k* interactions with the concept before time *t*+1, we use the all-zero embeddings to compensate. Before reshaping the embeddings to form a tensor, we take into account the forgetting curve hypothesis (Ebbinghaus, 2013), which states that the human memory retention declines over time, and thus give a different weight to each embedding according to its time gap to *t*+1. The simplest way of simulating the hypothesis is to use an exponential decay function (Woźniak et al., 1995). Therefore, we propose the following equation to transform the values in the *k* embeddings:


(3) $$\begin{array}{l} {\tilde{\text{x}}}_{i}=\exp\left(-\frac{\Delta t_{i}}{\theta }\right)\times {\tilde{\text{x}}}_{i}, \end{array}$$

Where Δ*t*_*i*_ is the time gap between interaction ${\tilde{\text{x}}}_{i}$ and time *t*+1, θ is a learnable parameter which controls the rate of decay. As such the interactions in the long past have small impacts on the current knowledge state. Then we reshape each of the *k* embeddings into a matrix (feature map) with shape *H* × *W*, where *H* × *W* = *d*_*e*_. In CAKT, we set *H* = *W*, but in practice one may set it to any shape as long as the equation holds. We stack the *k* matrices in their original chronological order and form a three-dimensional tensor of shape *k* × *H* × *W*. Then we feed the tensor into the 3D-ConvNets module which consists of four BasicBlocks. The architecture of BasicBlock is depicted in Figure 2B and Section 4.2. The 3D-ConvNets module outputs a tensor with the same shape as the input tensor, followed by a global average pooling layer to squash the output tensor in the time dimension into a matrix of shape *H* × *W*. Finally, the squashed matrix is stretched into the latent vector **m**_*t*_ of size *d*_*e*_. Without loss of generality, we set *d*_*e*_ = *d*_*h*_ so that **m**_*t*_ and **h**_*t*_ have the same length. The depth *k* of the tensor and the embedding size *d*_*e*_ (*d*_*h*_) are the two hyperparameters to be tuned.

Now we obtain two hidden state vectors **h**_*t*_ and **m**_*t*_, representing the student's overall latent knowledge state and the latent knowledge state on concept *c* covered by *q*_*t*+1_, respectively. In order to integrate the two features, we borrow the idea of the threshold mechanism in LSTM/GRU and propose a fusion gate to adaptively fuse them. The fusion gate outputs the hidden state ${\tilde{\text{h}}}_{t}$ at each step *t*, which is fed as input to the second LSTM layer (the LSTM on the right) in the figure. The second LSTM layer finally outputs a prediction vector **y**_*t*_ with length *M*, each element of which represents the probability that the student has mastered the corresponding knowledge concept at time *t*. Similarly to the first LSTM layer, we omit in the figure all LSTM units except the one of step *t* for the sake of simplicity. The predicted response to the next question *q*_*t*+1_ can be directly read from the element in **y**_*t*_ corresponding to the concept covered by *q*_*t*+1_.

### 4.2. The 3D-ConvNets module

The 3D-ConvNets module takes as input the three-dimensional tensor with shape *k* × *H* × *W*, which wraps the information in the *k* most recent interactions with the same concept covered by *q*_*t*+1_. We design a block named *BasicBlock* as shown in Figure 2B, and stack four BasicBlocks to form the 3D-ConvNets.

A BasicBlock consists of two three-dimensional convolutional layers, each of which is followed by a batch normalization layer and a ReLU layer. To facilitate the training, we use a residual connection to sum the input to BasicBlock and the output before the second ReLU layer. To ensure the input and output have the same shape, we use a three-dimensional filter with size 1 × 1 × 1 to convolve the input, which is depicted as the Downsample component in Figure 2B. The residual connections force the BasicBlocks to learn the residual features (He et al., 2016) from the input tensors, thereby facilitating the network optimization.

**Figure 3:**
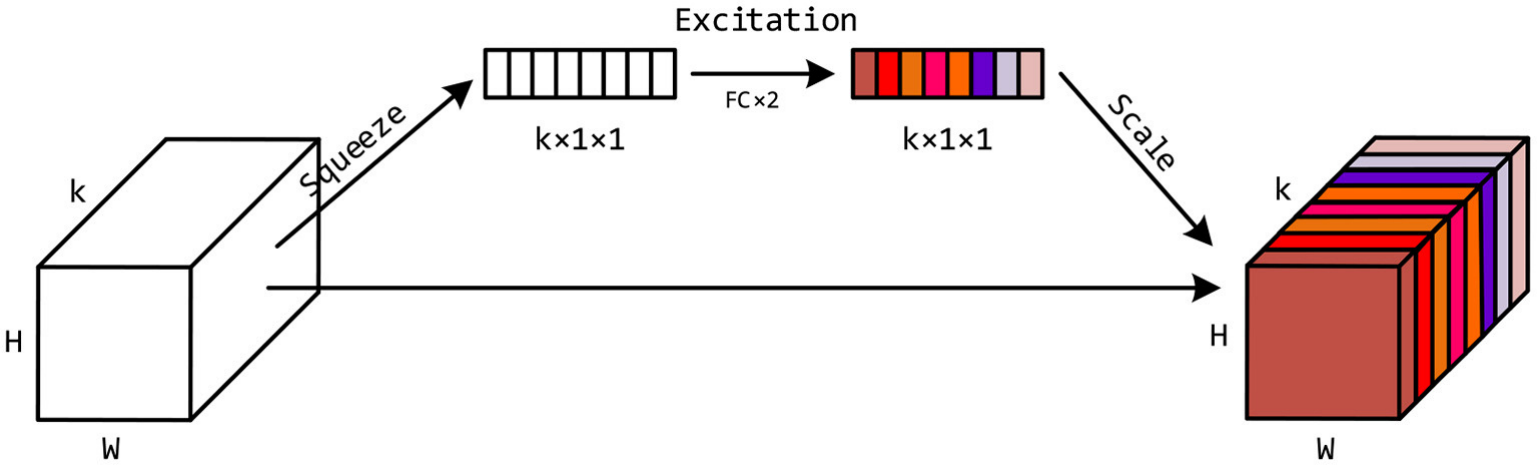
The architecture of the Timely-Squeeze-and-Excitation (TSE) layer. The squeeze stage uses global pooling to transform the input tensor with shape *k* × *H* × *W* into a vector with length *k*. The excitation stage uses two fully-connected layers to transform the *k* entries in the vector into values between 0 and 1. The scale stage uses these *k* values as importance weights to multiply with corresponding feature matrices in the input tensor to TSE.

In addition to the exponential decay function applied to the input embeddings, we want the 3D-ConvNets to further adaptively learn the importance of the feature maps at different time steps. Inspired by the Squeeze-and-Excitation Networks (Hu et al., 2018), we design a Timely-Squeeze-and-Excitation (TSE) layer and put it right after the second batch normalization layer in each BasicBlock. The architecture of TSE is shown in Figure 3. For the sake of simplicity, we ignore the batch and the channel dimension of the tensors. The input tensor to TSE with shape *k* × *H* × *W* is squeezed into a vector of length *k* using global pooling. Then we employ two fully-connected layers for excitation, where the first layer transforms the squeezed vector to a vector of length $\frac{k}{2}$ and uses ReLU for activation, and the second layer converts the vector back to length *k* and uses the Sigmoid function for activation. Each entry of the excited vector has a value between 0 and 1, which indicates the importance of the corresponding feature matrix in the input tensor to TSE. Finally, we scale the input tensor to TSE by multiplying each feature matrix with its importance weight, and form a new tensor with shape *k* × *H* × *W*, as depicted in the rightmost part of Figure 3.

In total, the BasicBlock takes as input a three-dimensional tensor with shape *k* × *H* × *W* and outputs a tensor with the same shape. We stack four BasicBlocks to form the 3D-ConvNets module. In the experiments we find that using small filters yields better results. Hence, following the principles in VGG (Simonyan and Zisserman, 2014) and FCN (Long et al., 2015), we use filters with size 3 × 3 × 3 in all the BasicBlocks and discard the pooling layers. We also use stride size with 1 and perform one zero-padding to ensure the tensor size unchanged. The filter size and the input/output shape in each BasicBlock are presented in Table 1.

**Table 1:** The filter size and the input/output shape in the BasicBlocks.

**Layer name**	**Output size**	**Filter size**	**In channel**	**Out channel**
BasicBlock1	*k*×H × W	3 × 3 × 3 3 × 3 × 3	1 4	4 4
BasicBlock2	*k*×H × W	3 × 3 × 3 3 × 3 × 3	4 8	8 8
BasicBlock3	*k*×H × W	3 × 3 × 3 3 × 3 × 3	8 4	4 4
BasicBlock4	*k*×H × W	3 × 3 × 3 3 × 3 × 3	4 1	1 1

After the convolution operations, we use a global average pooling layer to squash the output tensor on the time dimension into a matrix ${\hat{\text{m}}}_{t}$ of shape *H* × *W*, which can be formulated as follows:


(4) $$\begin{array}{l} {\hat{\text{m}}}_{t}=\frac{1}{k}\overset{k}{\underset{i=1}{\sum }}{\text{m}}^{i}, \end{array}$$

Where *k* is the depth of the output tensor and **m**^*i*^ is the *i*^*th*^ feature map in the output tensor. Then ${\hat{\text{m}}}_{t}$ is further stretched into a hidden vector **m**_*t*_ of size *d*_*e*_, which represents the latent knowledge state on the concept covered by *q*_*t*+1_.

### 4.3. Adaptive feature fusion

In order to fuse **h**_*t*_ and **m**_*t*_, we borrow the idea from the threshold mechanism in LSTM/GRU and propose a fusion gate to adaptively learn the weights of the features. The weights control how much information of the two latent features should be preserved. The process can be formulated as follows:


(5) $$\begin{array}{l} {\text{z}}_{t}^{1}=\sigma \left(\left[{\text{m}}_{t}⊕{\text{h}}_{t}\right]{\text{W}}_{z}^{1}+{\text{b}}_{z}^{1}\right), \end{array}$$

(6) $$\begin{array}{l} {\text{z}}_{t}^{2}=\sigma \left(\left[{\text{m}}_{t}⊕{\text{h}}_{t}\right]{\text{W}}_{z}^{2}+{\text{b}}_{z}^{2}\right), \end{array}$$

(7) $${\tilde{h}}_{t}=z_{t}^{1}⊙m_{t}+z_{t}^{2}⊙h_{t},$$

Where ⊕ represents concatenating two vectors, ${\text{W}}_{z}^{1}$ and ${\text{W}}_{z}^{2}$ are two weight matrices with shape 2*d*_*e*_×*d*_*e*_, ${\text{b}}_{z}^{1}$ and ${\text{b}}_{z}^{2}$ are two bias vectors with length *d*_*e*_, σ represents the Sigmoid function and ⊙ represents the Hadamard product between two vectors. Generally speaking, we concatenate **m**_*t*_ with **h**_*t*_ into a vector with length 2*d*_*e*_, and then use two fully-connected layers with Sigmoid activation to transform the vector into two gates ${\text{z}}_{t}^{1}$ and ${\text{z}}_{t}^{2}$, which are shown in Equations (5), (6). The two gates control the information preserved in **m**_*t*_ and **h**_*t*_, respectively. We can then fuse the two features into a single feature ${\tilde{\text{h}}}_{t}$, as shown in Equation (7).

Finally, the second LSTM layer takes ${\tilde{\text{h}}}_{t}$ as input and outputs the predicted knowledge state vector **y**_*t*_ with length *M*, where each element represents the probability that the student has mastered the corresponding knowledge concept at time *t*.

### 4.4. Objective function

The objective function is a binary cross-entropy loss function, calculated using the predicted probability *p*_*t*_ that *q*_*t*_ is correctly answered and the ground-truth response *a*_*t*_, for all time step *t*. As we discussed in the end of Section 4.1, *p*_*t*_ can be directly read from the element in **y**_*t*−1_ corresponding to the concept in *q*_*t*_. The function can be formulated as:


(8) $$\begin{array}{l} L=-\underset{t}{\sum }\left(a_{t}\text{ log }p_{t}+\left(1-a_{t}\right)\text{log}\left(1-p_{t}\right)\right) \end{array}$$

As inference, we set 0.5 as the threshold probability for the predicted responses, i.e., the answer to *q*_*t*_ is predicted as correct if *p*_*t*_ ≥ 0.5, and is predicted as incorrect otherwise.

## 5. The CECAKT model

The CAKT model uses a global average pooling layer to squash the tensor output by the 3D-ConvNets module and produces the feature map representing the knowledge state on the concept of the next question, as depicted in Equation (4). This layer would ignore the relationships between the elements in the tensor and also cause information loss during feature aggregation (squashing of the tensor), i.e., the elements at the same position of different feature maps are simply averaged. The implication in knowledge tracing is that the previous latent knowledge states pertaining to the concept covered by the next question at different time steps are equally weighted to compute the latent state of the current step, so that the differences in contributions of latent knowledge states at different steps are lost.

**Figure 4:**
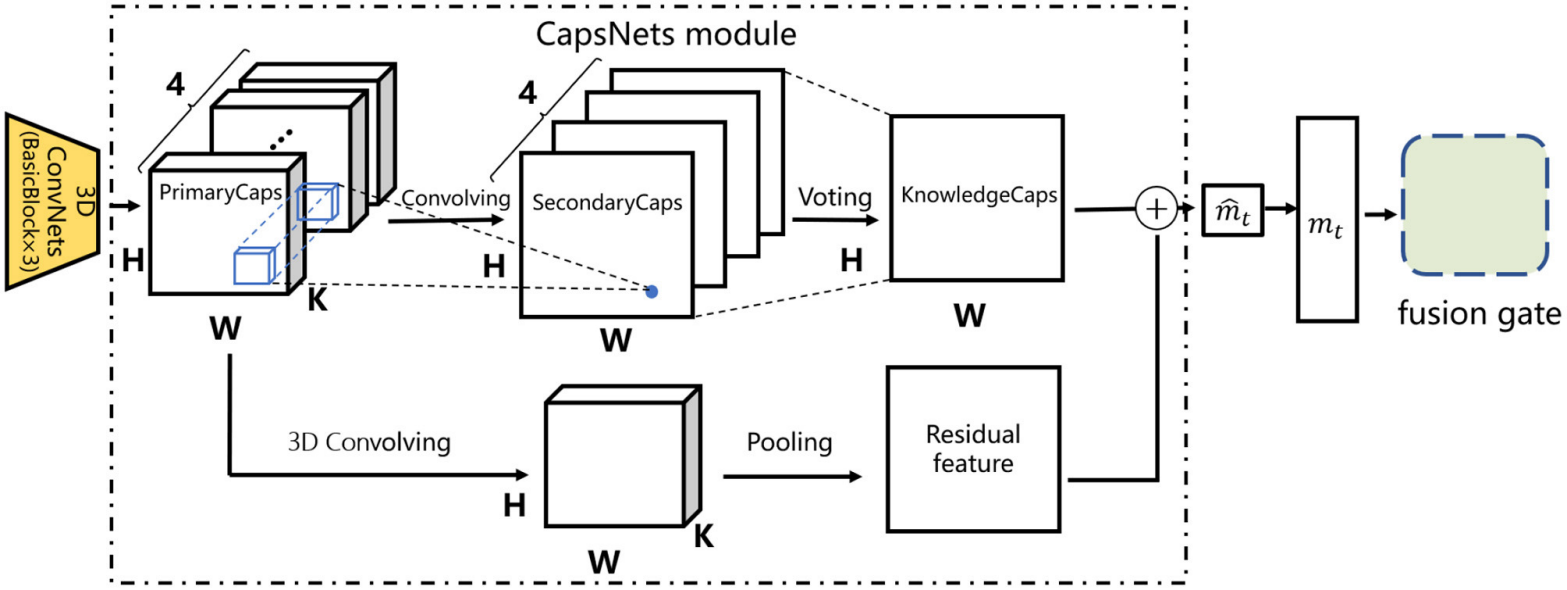
The architecture of the CapsNets module in the CECAKT model.

For more accurate modeling, we further propose to replace the global average pooling layer with capsule networks (Sabour et al., 2017) and obtain the CECAKT model. In order not to make CECAKT a complex model, we design a simple CapsNets module as follows. Specifically, we discard the BasicBlock4 layer in CAKT and view the output of the BasicBlock3 layer as a group of primary capsules (PrimaryCaps), i.e., 4 channels of *k*D capsules, where each channel is an *H* × *W* grid consisting of the same type of capsules. Then we use four two-dimensional convolutional filters of size 3 × 3 to transform the PrimaryCaps into a group of secondary capsules (SecondaryCaps), which still contain 4 types of capsules organized in 4 *H* × *W* grids. However, the capsules are convolved into 1D (i.e., a scalar), where each 1D secondary capsule captures the relationships among a group of PrimaryCaps within the same area across the 4 channels. Note that we set the number of PrimaryCaps types and the number of SecondaryCaps types both to 4 for simplicity. Finally, we adopt a voting (dynamic routing) layer to transform the SecondaryCaps into a group of knowledge capsules (KnowledgeCaps), which have only one type of 1D capsules in one *H* × *W* grid, representing whether the corresponding knowledge concept is mastered or not. Particularly, each knowledge capsule is a weighted aggregation of the SecondaryCaps at the same position across the 4 channels, i.e., along the time dimension, according to the trainable coefficients of the SecondaryCaps. Compared to performing average pooling along the time dimension in CAKT, the voting mechanism reduces information loss by preserving the differences in contributions of the SecondaryCaps at different time steps. For knowledge tracing, this implies that the previous latent knowledge states pertaining to the concept covered by the next question contribute adaptively to the computation of the latent state at the current step. The entire process is depicted in the upper half of Figure 4.

Specifically, the voting layer works as follows. Denote by $u_{i}^{t}$ a capsule *i* in the SecondaryCaps, where 0 ≤ *i*<*H*×*W* and 1 ≤ *t* ≤ 4 is the capsule type. The input *s*_*i*_ to the capsule *i* in the KnowledgeCaps is calculated as follows:


(9) $$\begin{array}{l} {û}_{i}^{t}=w^{t}\times u_{i}^{t}, \end{array}$$

(10) $$\begin{array}{l} s_{i}=\underset{t}{\sum }c_{i}^{t}{û}_{i}^{t}, \end{array}$$

Where *w*^*t*^ is the weight shared among all the capsules of type *t*, ${û}_{i}^{t}$ is the so-called “prediction vector” (1D for the SecondaryCaps), and $c_{i}^{t}$ is the routing coefficient of ${û}_{i}^{t}$. The routing coefficients are determined by the dynamic routing algorithm, which is usually computed as a “routing softmax”:


(11) $$\begin{array}{l} c_{i}^{t}=\frac{\text{exp}\left(b_{i}^{t}\right)}{\sum_{l=0}^{H\times W}\text{exp}\left(b_{l}^{t}\right)}, \end{array}$$

Where $b_{i}^{t}$ is the log prior probability that ${û}_{i}^{t}$ should be routed to the capsule *i* in the KnowledgeCaps and is learned during optimization. Finally, the capsule *v*_*i*_ in the KnowledgeCaps is calculated using a non-linear “squashing”:


(12) $$\begin{array}{l} v_{i}=\frac{||s_{i}|{|}^{2}}{1+||s_{i}|{|}^{2}}\frac{s_{i}}{\left||s_{i}|\right|}, \end{array}$$

Where *s*_*i*_ is a 1D vector in our case.

In this way, the tensors output by the 3D-ConvNets module are transformed into a two-dimensional feature map through dynamic routing, where the important elements in each input feature map and their relationships are preserved in the final map. To facilitate training, we also add a residual link from the PrimaryCaps to the KnowledgeCaps. The link uses a 3D convolutional layer to process the PrimaryCaps, followed by an average pooling layer to obtain a 2D residual feature map. This is depicted in the lower half of Figure 4. The two feature maps are added together to obtain the map ${\hat{\text{m}}}_{t}$, which was originally calculated using average pooling in CAKT as depicted in Equation (4). The entire structure bounded in the dashed rectangle is referred to as the CapsNets module. The rest of the architecture in CECAKT is exactly the same as that of CAKT.

## 6. Experiments

### 6.1. Datasets

We perform empirical evaluation on four real-world datasets that are commonly used in literature (Abdelrahman and Wang, 2019; Ghosh et al., 2020; Shen et al., 2020; Long et al., 2022). The statistics of the datasets are shown in Table 2.

- **ASSISTments2009**^2^: This dataset is gathered in the school year 2009–2010 from the ASSISTments education platform. We use the skill builder data of ASSISTments2009, which consists of 110 distinct questions (knowledge concepts), 4,151 students and 325,637 exercise records (student-question interactions).
- **ASSISTments2015**^3^: This dataset is collected in 2015. It is an updated variant to ASSISTments2009. It includes 100 distinct questions, 19,840 students and 683,801 exercise records. This dataset has the largest number of students, but the average number of exercise records per student (34) is the smallest among the four datasets.
- **ASSISTments2017**^4^: This dataset is collected from the ASSISTments education platform in 2017. It includes 102 distinct questions, 1,709 students and 942,816 exercise records
- **Statics2011**^5^: This dataset is collected from a statistics course at Carnegie Mellon University in the fall of 2011. It contains 1,223 distinct questions, 333 students, and 189,297 exercise records.

**Table 2:** Statistics of the datasets.

**Dataset**	**#Questions (#Concepts)**	**#Students**	**#Interactions (#Exercises)**	**#Interactions per student**
ASSISTments2009	110	4,151	325,637	78
ASSISTments2015	100	19,840	683,801	34
ASSISTments2017	102	1,709	942,816	552
Statics2011	1,223	333	189,297	568

Among these datasets, ASSISTments2017 and Statics2011 have much longer interaction sequences than the two other datasets. So following the methods in the related work (Piech et al., 2015; Zhang et al., 2017), we conduct a fold operation on the two datasets. In particular, when the length of a sequence exceeds 200, we split the sequence into sub-sequences so that the length of each sub-sequence is less than or equal to 200.

### 6.2. The hyperparameters and network instance of CAKT and CECAKT

The important hyperparameters for tuning are the depth of the input tensor *k*, the embedding size of the input interaction *d*_*e*_, the hidden state size of the first LSTM layer *d*_*h*_ and the number of capsule types in the PrimaryCaps and SecondaryCaps *n*. To facilitate the fusion of **m**_*t*_ and **h**_*t*_, we set *d*_*e*_ = *d*_*h*_. Also, the reshaped interaction matrix satisfies *H* × *W* = *d*_*e*_, where *H* and *W* are the height and width. Without loss of generality, we set *H* = *W* and therefore *d*_*e*_ must be the square of an integer value. Nevertheless, one may set *H* not equal to *W* as long as *H* × *W* = *d*_*e*_ holds. As such we just need to tune *k*, *H* and *n* in the training phase. After tuning, we set *k* = 6, *H* = 17 for CAKT and set *k* = 4, *H* = 15 and *n* = 4 for CECAKT (on ASSISTments2017, *n* = 8), to obtain the main results in Section 6.5.1. We also present the results when varying the hyperparameters of CECAKT in Section 6.6.

The 3D-ConvNets module in CAKT is constructed by stacking four BasicBlocks, each of which contains a Conv-BN-ReLU layer and a Conv-BN-TSE-ReLU layer with a residual link. We set the filter size of the convolutional layers to 3 × 3 × 3 as discussed in Section 4.2, and vary their channel sizes in the forward pass. The sizes of the BN and ReLU layers are decided by the convolutional layers. The two LSTM layers use the same hidden state size. The 3D-ConvNets module in CECAKT is constructed by stacking three BasicBlocks. The filter size of the convolutional layer transforming the PrimaryCaps into SecondaryCaps is set to 3 × 3.

### 6.3. The comparative models

We compare our models with eight representative deep models for the knowledge tracing task, namely DKT (Piech et al., 2015), DKVMN (Zhang et al., 2017), SKVMN (Abdelrahman and Wang, 2019), SAKT (Pandey and Karypis, 2019), EKT (Huang et al., 2019), CKT (Shen et al., 2020), DKT-F (DKT+forgetting) (Nagatani et al., 2019), and AKT (Ghosh et al., 2020). Among the models, EKT has two variants, which employ the Markov chain and attention mechanism, respectively. We choose the variant using attentions since it is reported to perform better. One issue is that EKT requires the text information of questions as input. Since the datasets do not contain text information, we slightly modify the input features of EKT and obtain two variants for comparison. The first one uses a fixed randomized embedding to represent the text information for each distinct question, which is referred to as EKT-R. The second one replaces the randomized text information embedding with the knowledge concept embedding at each time step, which is referred to as EKT-C. The AKT model has also two variants in Ghosh et al. (2020), namely AKT-R and AKT-NR. AKT-R requires both knowledge ID and question ID as input, whereas AKT-NR only requires knowledge ID as other models do. For fair comparison, we choose the AKT-NR model since all other models do not use the question ID information. In addition, the datasets except ASSISTments2009 and ASSISTments2017 do not contain the information of question ID (Ghosh et al., 2020). We reimplement SKVMN and DKT-F since we have not found the released source code. We use the source code on GitHub for DKT ^6^, DKVMN ^7^, EKT ^8^, CKT ^9^ and AKT ^10^. We obtain the code for SAKT from the original authors.

### 6.4. Model training and the evaluation metric

We implement CAKT and CECAKT using Pytorch 1.6 and train them on an NVIDIA Tesla-V100 card with 16GB memory. We use the Adam optimizer to optimize the network parameters. We set the L2 regularization term to 5e-5 and the initial learning rate to 0.001, with a decay of 0.3 every 5 epochs. The source code is available at https://github.com/Badstu/CAKT.

Similarly to the settings in Piech et al. (2015), Zhang et al. (2017), Abdelrahman and Wang (2019), and Ghosh et al. (2020), we use 20% of the interaction sequences to form a testing set for each dataset, and split the remaining sequences into five folds for cross validation. For each dataset, the hyperparameters of each model are determined when it has the best average performance on the validation sets. Then we report the corresponding average results on the testing set for each model on each dataset. The evaluation metric on the testing set is the Area Under the ROC Curve, referred to as AUC (Belohradsky et al., 2011), which is commonly used to evaluate the performance of knowledge tracing models (Piech et al., 2015; Zhang et al., 2017; Abdelrahman and Wang, 2019; Ghosh et al., 2020). When AUC=0.5, it means that the prediction makes no difference from a random guess. The higher value the AUC, the better the prediction performance of the model. We also compute the statistical significance of the results, verified using a 2-tailed Student's t-test.

### 6.5. The results of comparative evaluation

#### 6.5.1. Main results

The main comparative results are reported in Table 3. For each row, we use the bold font for the results of CAKT and CECAKT if they both perform better than the comparative models. Otherwise, we use the bold font only for the best result in each row. We observe that CAKT performs better than the comparative models on three datasets, namely, ASSISTments2009, ASSISTments2017 and Statics2011. On ASSISTments2015, CAKT performs slightly worse but still very comparable with CKT and AKT-NR. On the other hand, CECAKT further improves CAKT and performs the best on all the four datasets. The results prove the effectiveness of using the three-dimensional convolutional networks to model the learning curve theory, and the effectiveness of replacing the average pooling layer with the capsule networks to achieve more accurate modeling.

**Table 3:** The test AUC results (%) of all the models.

**Dataset**	**DKT**	**DKVMN**	**SKVMN**	**SAKT**	**EKT-R**	**EKT-C**	**DKT-F**	**CKT**	**AKT-NR**	**CAKT**	**CECAKT**
ASSIST2009	81.19	80.02	67.39	76.59	76.46	76.45	81.88	82.13	81.84	82.37*	82.85*
ASSIST2015	71.95	72.33	67.01	73.27	70.65	70.35	72.96	73.45	73.43	73.31	**73.45**
ASSIST2017	64.47	68.53	56.95	64.85	60.25	61.56	73.48	72.16	72.06	73.68*	74.41*
Statics2011	79.00	80.42	78.41	81.43	75.65	77.73	82.76	82.38	82.74	**82.78**	83.41*

The results of CAKT and CECAKT are in the last two columns. For each row, CAKT and CECAKT are both bolded if they perform both better than the comparative models. Otherwise, the best result in each row is bolded. An asterisk (*) indicates *p* < 0.05 in the significance test.

By analyzing the results in detail, we notice that the improvements of CAKT and CECAKT over the comparative models on ASSISTments2009 and ASSISTments2017 are more significant than that on the other two datasets. To explore the reason behind, we calculate the average number of repeated practices on the same knowledge concept per student in the four datasets. The results are shown in Table 4. We observe that on ASSISTments2009 and ASSISTments2017, students repeatedly practice 11 and 13.2 times on average for the same knowledge concept, respectively. As such the 3D-ConvNets module and the CapsNets module could learn enough information from a student's past practices on a concept when predicting the next response to the concept. Therefore, on the two datasets, the improvements of CAKT and CECAKT over other models are larger. In contrast, ASSISTments2015 and Statics2011 have a small average number of repeated practices, which are 5.4 and 3.2 per student, respectively. As a result, the 3D-ConvNets module and the CapsNets module may not learn enough information about a student's past experience on applying the knowledge concept covered by the question to be answered at each step, which in turn leads to smaller prediction performance improvements on the two datasets. The results indeed confirm the importance of explicitly modeling the learning curve theory, especially when there are a sufficient number of past practices on the same knowledge concept.

**Table 4:** The average number of repeated practices on the same knowledge concept per student.

	**ASSISTments2009**	**ASSISTments2015**	**ASSISTments2017**	**Statics2011**
# repeated practices	11.0	5.4	13.2	3.2

Furthermore, it is worth noting that CAKT and CECAKT greatly improve the performance of DKT on all datasets. Remember that the two models retain the LSTM structure used in DKT to learn from the entire sequence in the past. Therefore, the big improvements over DKT are mainly due to the explicit modeling of the learning curve theory. While this proves the importance of the modeling, more advanced structures may be used to replace LSTM and further boost the prediction performance.

#### 6.5.2. Convergence rate

**Figure 5:**
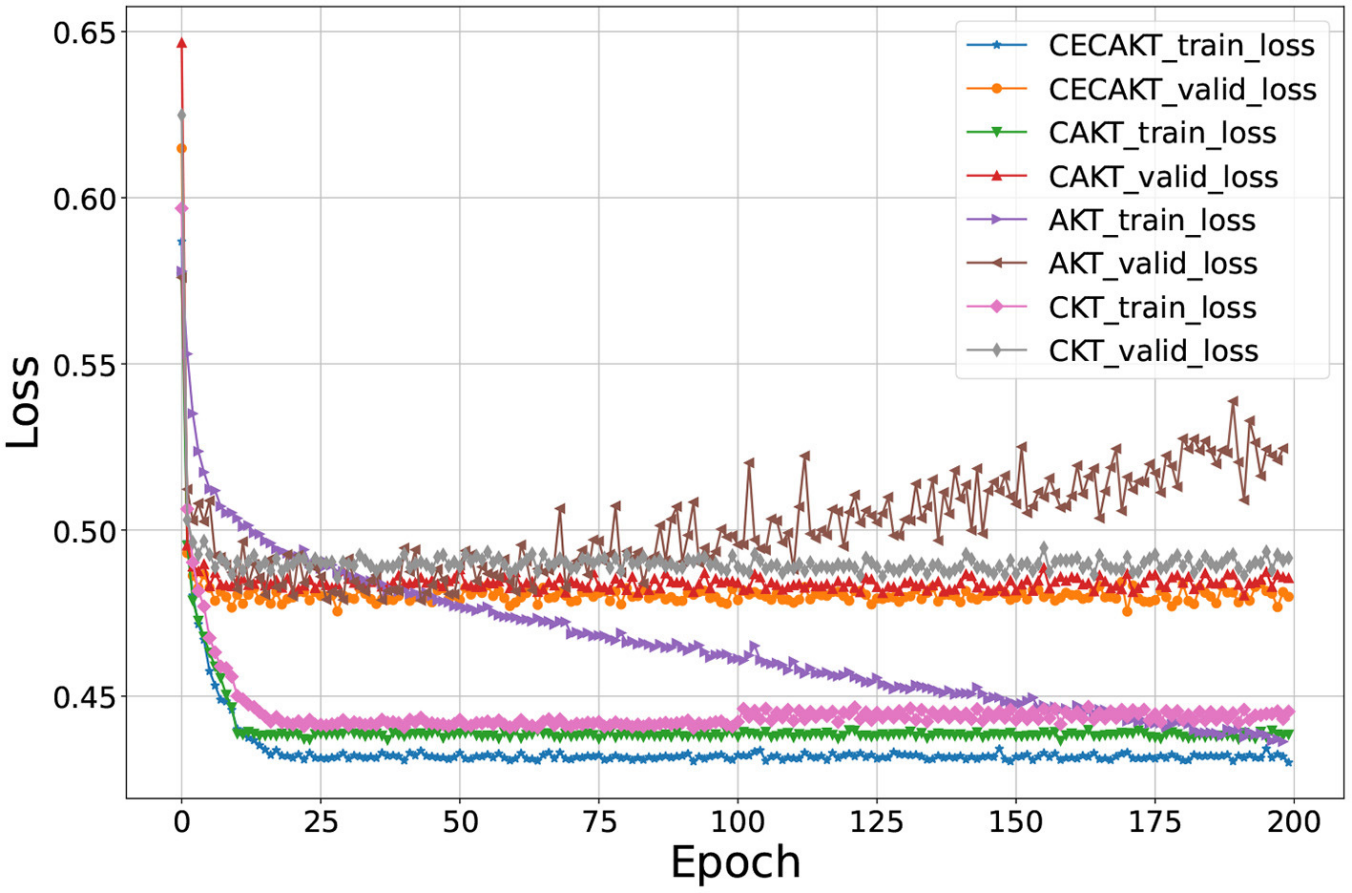
Convergence rate. The horizontal axis represents the number of epochs, and the vertical axis represents the loss values.

In Table 3 we observe CKT and AKT-NR have the overall closest performance to that of CAKT and CECAKT. In this section, we further compare the convergence rates of the four models and show the results on the ASSISTments2009 dataset in Figure 5. The results on the other datasets are similar and thus omitted. However, they are available upon request. We use the hyperparameters tuned in the main results for each model. In particular, we set *k* = 6 and *H* = 17 for CAKT, and set *k* = 4, *H* = 15 and *n* = 4 for CECAKT. The initial learning rate is 0.001 with decay of 0.3 every 5 epochs, and the L2 regularization term is 5e-5. Both CKT and AKT-NR use learning rate 0.001. AKT-NR sets the L2 regularization to 1e-5 and CKT does not have a regularization item. In the figure, we observe that our CAKT and CECAKT model both converge within 10 epochs, which are faster than CKT and AKT-NR. CKT converges in about 20 epochs. AKT-NR does not converge even after training for 200 epochs and becomes over-fitting after 75 epochs since the validation loss turns to increase gradually. In the original paper of AKT-NR (Ghosh et al., 2020), the authors perform early stopping to avoid over-fitting. In summary, CAKT and CECAKT converge much faster than CKT and AKT-NR, while achieving the better performance in terms of AUC. CECAKT has the lowest training loss and validation loss, and therefore performs the best in terms of AUC on the testing set.

### 6.6. Sensitivity analysis

In this part, we explore how the three hyperparameters discussed in Section 6.2 influence the performance of CECAKT. We conduct sensitivity analysis on the number of recent interactions *k* on the same knowledge concept, the height *H* of the input tensor to the 3D-ConvNets module (i.e., the embedding size of the input interaction *d*_*e*_ since we set *H* = *W* and *d*_*e*_ = *H* × *W*), and the number of capsule types *n* in the PrimaryCaps and SecondaryCaps. For each experiment, we fix two hyperparameters and adjust the remaining one.

The first experiment focuses on the hyperparameter *k*, that is, how many past interactions on the same knowledge concept are needed for capture the experience. We fix *H* = 15 and *n* = 4, and vary *k* from 4 to 10 with increments 2. The results on each dataset are reported in Table 5. We observe that the performances are relatively stable and a moderate *k* value can already bring the overall best results. This is reasonable because the most recent interactions have the highest impact on the current knowledge state, according to the forgetting curve hypothesis (Ebbinghaus, 2013). Particularly, when *k* = 4, CECAKT achieves the best performance on ASSISTments2009, ASSISTments2015 and Statics2011. On ASSISTments2017, although the best performance is obtained at *k* = 10, the results for different values of *k* are quite close. The moderate value of *k* indicates that CECAKT just needs a small 3D-ConvNets module, which reduces the complexity of the model.

**Table 5:** The AUC results (%) for varying *k*.

**H&n**	***k***	**ASSISTments2009**	**ASSISTments2015**	**ASSISTments2017**	**Statics2011**
*H* = 15	*k* = 4	**82.85**	**73.45**	74.25	**83.41**
*n* = 4	*k* = 6	82.75	73.41	74.25	83.19
	*k* = 8	82.74	73.39	74.30	83.30
	*k* = 10	82.63	73.34	**74.31**	83.34

The best AUC in each column is bolded.

The second experiment focuses on the height of the input tensor to the 3D-ConvNets module *H*. We fix *k* = 4 and *n* = 4, and vary the value of *H* from 11 to 17 with increments 2. In CECAKT we set *H* = *W* and *H* × *W* = *d*_*e*_ = *d*_*h*_, thus *H* decides the size of the feature map, the input embedding size and the hidden vector size of the LSTM layers and the CapsNets module. The results are reported in Table 6. We observe that overall a bigger *H* brings better results, since big feature maps or long vectors usually contain more information. However, the values of *H* are still moderate to achieve the best results on the four datasets, making the sizes of both the input tensor and the input embedding small.

**Table 6:** The AUC results (%) for varying *H*.

**k&n**	***H***	**ASSISTments2009**	**ASSISTments2015**	**ASSISTments2017**	**Statics2011**
*k* = 4	*H* = 11	82.74	73.32	**74.32**	83.25
*n* = 4	*H* = 13	82.76	73.37	74.30	83.25
	*H* = 15	**82.85**	**73.45**	74.25	**83.41**
	*H* = 17	82.75	73.41	74.24	83.19

The best AUC in each column is bolded.

The third experiment focuses on *n*, the number of capsule types in the PrimaryCaps and SecondaryCaps. We fix *H* = 15 and *k* = 4, and vary *n* from 4 to 16 with increments 4. The results are reported in Table 7. We observe that a small number of capsule types can already bring the best results. In particular, CECAKT achieves the best results at *n* = 4 on ASSISTments2009, ASSISTments2015 and Statics2011, and achieves the best result at *n* = 8 on ASSISTments2017. The small number of capsule types also prevents the size of the entire model from becoming too large.

**Table 7:** The AUC results (%) for varying *n*.

**H&k**	***n***	**ASSISTments2009**	**ASSISTments2015**	**ASSISTments2017**	**Statics2011**
H=15	*n* = 4	**82.85**	**73.45**	74.25	**83.41**
k=4	*n* = 8	82.83	73.38	**74.41**	83.40
	*n* = 12	82.81	73.31	74.25	83.37
	*n* = 16	82.84	73.31	74.22	83.37

The best AUC in each column is bolded.

### 6.7. Ablation study

In the first paragraph of Section 1.2, we enumerate the possible choices of the architecture to capture the knowledge state pertaining to the same concept, including RNNs/LSTM, self-attention networks and CNNs, and discuss the shortcomings of these choices. In this section, we empirically demonstrate the effect of these alternative architectures. We do this by replacing the 3D-ConvNets module in CAKT with fully-connected networks, LSTM, self-attention and ordinary CNNs, respectively, and obtain four ablation models, namely, FC_KT, LSTM_KT, SA_KT and CNN_KT. The two-layer LSTM network is retained. Furthermore, we investigate the necessity of keeping the 3D-ConvNets module in CECAKT, i.e., whether we can use the capsule networks solely in CECAKT. We do this by removing the 3D-ConvNets module and feeding the input tensor directly into the CapsNets module. The resulted model is referred to as CEKT.

The results are reported in Table 8. We observe that the CAKT model performs much better on all the datasets than the corresponding ablation models. This justifies the design choice of the 3D-ConvNets module and may verify our conjecture in the third paragraph of Section 1.2, i.e., the reason that 3D-ConvNets are the better choice for modeling the learning curve theory. On the other hand, if the 3D-ConvNets module is discarded from CECAKT, the performance of CEKT significantly decreases on all the four datasets. The results show that the 3D-ConvNets module is still a necessary component in CECAKT, and that the CapsNets module mainly tackles the information loss problem induced by the pooling layer.

**Table 8:** The AUC results (%) of ablation study.

**Ablation models**	**ASSISTments2009**	**ASSISTments2015**	**ASSISTments2017**	**Statics2011**
FC_KT	81.01	71.73	72.22	79.55
LSTM_KT	80.57	71.63	71.82	79.23
SA_KT	80.46	71.56	72.32	79.56
CNN_KT	81.17	71.62	73.04	79.64
CAKT	82.37*	73.31*	73.68*	82.78*
CEKT	80.68	73.02	72.43	79.47
CECAKT	82.85*	73.45*	74.41*	83.41*

An asterisk (*) indicates *p* < 0.05 in the significance test. The results of CAKT and CECAKT are in bold because they both perform better than their corresponding ablation models.

### 6.8. The impact of learning curve modeling

**Figure 6:**
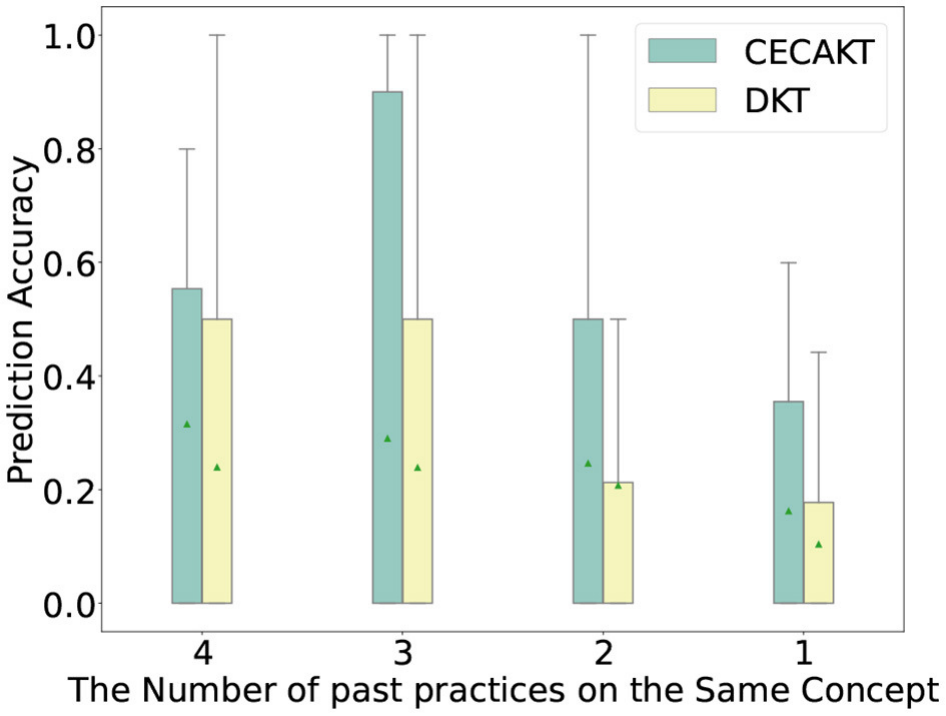
Prediction accuracy for interactions with different number of past practices on the same knowledge concept, CECAKT Vs. DKT.

**Figure 7:**
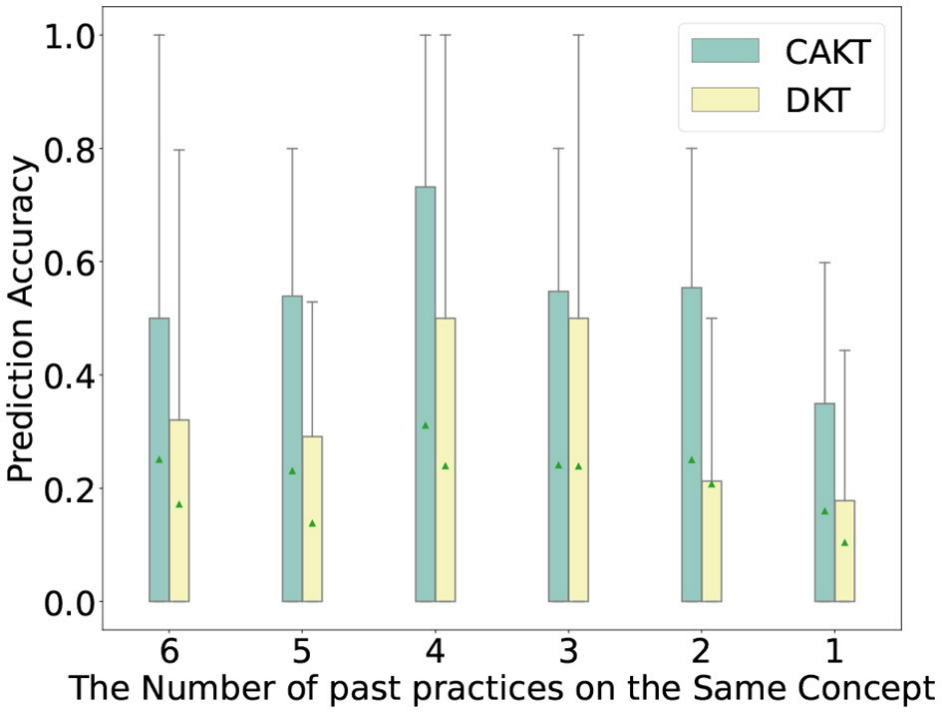
Prediction accuracy for interactions with different number of past practices on the same knowledge concept, CAKT Vs. DKT.

In this section, we study how the modeling of learning curves impacts the performance of the proposed model. We first plot the prediction accuracy of student-question interactions under different numbers of past practices on the same knowledge concept. In particular, we extract the interactions from all the four datasets such that the corresponding student has previously practiced on the concept of the question for 1, 2, 3, and 4 times, respectively, and divide the interactions into four categories based on the number of past practices. Then for each category of interactions, we run CECAKT to predict the student responses, based on which we calculate the statistics of prediction accuracy for each category. We set 4 as the maximum number of past practices for CECAKT because the model sets the hyperparameter *k* = 4, i.e., it only considers 4 past practices on the same knowledge concept maximally. We do the same for CAKT, except that the maximum number of past practices is set to 6 (because we set *k* = 6 for CAKT). The results are plotted in Figures 6, 7. We plot the results of DKT for comparison since the only different between DKT and CAKT/CECAKT is whether the learning curve theory is modeled. We observe that for each category of interactions, CECAKT and CAKT both have higher average and third-quartile prediction accuracy compared to DKT. The results prove that the modeling of learning curves can increase the prediction accuracy when there are multiple past practices on the same knowledge concept.

**Figure 8:**
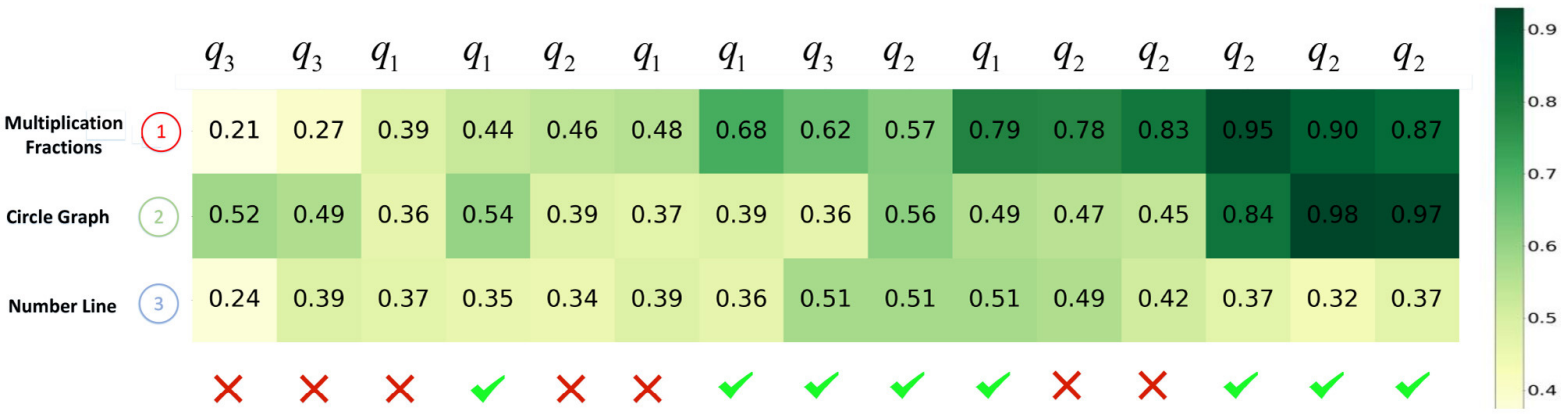
The evolution of predicted knowledge states produced by CECAKT. The y-axis represents the knowledge concepts, and the x-axis represents the student's responses to a sequence of the questions. The grids represent the predicted knowledge states, i.e., the predicted probabilities that the student has mastered the knowledge concepts.

**Figure 9:**
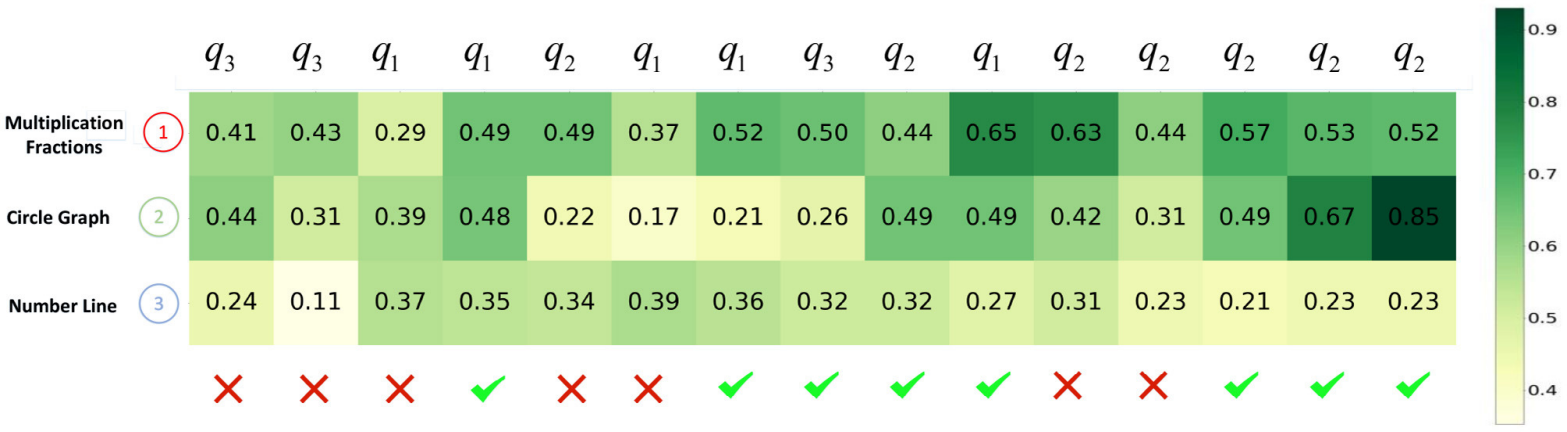
The evolution of predicted knowledge states produced by DKT. The y-axis represents the knowledge concepts, and the x-axis represents the student's responses to a sequence of the questions. The grids represent the predicted knowledge states, i.e., the predicted probabilities that the student has mastered the knowledge concepts.

Second, we visualize the impact of learning curve modeling through a use case. In Figures 8, 9, we plot the evolution of a student's knowledge state when she interacts with a sequence of questions, predicted by CECAKT and DKT, respectively. In the figures, *q*_1_, *q*_2_ and *q*_3_ are the questions related to the knowledge concept *Multiplication Fractions, Circle Graph* and *Number Line*, respectively, which are shown on the y-axis. In other words, each *q*_1_ denotes a distinct question pertaining to *Multiplication Fractions*, and the similar notation applies to *q*_2_ and *q*_3_. The x-axis shows the student's true responses to the questions, i.e., correct or incorrect answers. Each row of grids show the evolution of predicted mastery probability of the corresponding concept. Comparing the two figures, we may find that CECAKT predicts the knowledge states much more accrurately than DKT, when the same knowledge concept is involved in the past interactions. Take *q*_2_ for example, the corresponding questions are related to the concept *Circle Graph*. At the first time that the student answers *q*_2_, the mastery probabilities of the concept predicted by CECAKT and DKT are 0.39 and 0.22, respectively. Then for the second time that she answers *q*_2_ (a different question from the first *q*_2_, which also contains the concept *Circle Graph*), the mastery probability predicted by CECAKT is 0.56, whereas the mastery probability predicted by DKT is 0.49. Remember that we set 0.5 as the threshold probability for the predicted responses (correct or incorrect answers). Since the student correctly answers the question, the prediction of CECAKT is right and the prediction of DKT is wrong. This is because CECAKT has taken the previous practice into account. For the last three answers to *q*_2_, CECAKT makes all the right predictions and the predicted mastery probabilities are very high. On the contrary, DKT makes one wrong prediction and the predicted mastery probabilities are fairly low. Similar results can be observed for *q*_1_ and *q*_3_. The observations suggest that modeling the learning curve theory can largely increase the accuracy of the predicted knowledge states.

## 7. Conclusion

In this paper, we propose two novel models CAKT and CECAKT for the knowledge tracing task. The models are motivated by the learning curve theory, which states more practices would bring more improvement on a skill and is observed in the real-life dataset. We discuss the possible architecture choices for explicitly modeling the learning curve theory. Based on the discussion, we design a module built with three-dimensional convolutional neural networks to model a student's latent knowledge state on the concept covered by the question to be answered, and design a module built with capsule networks to reduce information loss and further improve the modeling accuracy. On the other hand and without loss of generality, we employ an LSTM network to learn the student's overall knowledge state. We then design a fusion gate to fuse the two types of latent features, and use the fused feature to predict the student's response to the next question. As such, when predicting a student's response to the next question, we collectively consider her recent experience on applying the concept covered by the question and her overall experience on all the knowledge concepts. Experimental results show that our CAKT and CECAKT models outperform current representative models and their own variants for modeling the learning curves. The major improvements on the two datasets with a large number of repeated practices on the same concept further verify the effectiveness of the learning curve modeling.

While the current work proves the importance of explicit modeling of the learning curve theory, in the future we would investigate structures other than two-layer LSTM networks for capturing the overall knowledge state to further boost the KT performance. Also, an interesting question is what heterogeneous patterns the 3D-ConvNets module and the first LSTM layer have learned from the interaction sequences, respectively. We leave this for future work.

## Data availability statement

Publicly available datasets were analyzed in this study. This data can be found at: https://sites.google.com/site/assistmentsdata/home/2009-2010-assistment-data; https://sites.google.com/site/assistmentsdata/datasets/2015-assistments-skill-builder-data; https://sites.google.com/view/assistmentsdatamining; https://pslcdatashop.web.cmu.edu/DatasetInfo?datasetId=507.

## Author contributions

HS and XiL implemented the CECAKT model, conducted all the related experiments, and plotted all the related figures. SY implemented the CAKT model, conducted all the related experiments, and plotted all the related figures. The three authors together drafted the initial manuscript. XuL proposed the overall idea of the work and revised the manuscript. All authors contributed to the article and approved the submitted version.

## Footnotes

^1^We follow the convention that each question covers exactly one knowledge concept and all the questions covering the same concept are considered as a single question. Thus, the questions with the same concept have the same one-hot encoding.

^2^ https://sites.google.com/site/assistmentsdata/home/2009-2010-assistment-data

^3^ https://sites.google.com/site/assistmentsdata/datasets/2015-assistments-skill-builder-data

^4^ https://sites.google.com/view/assistmentsdatamining

^5^ https://pslcdatashop.web.cmu.edu/DatasetInfo?datasetId=507

^6^ https://github.com/mmkhajah/dkt

^7^ https://github.com/jennyzhang0215/DKVMN

^8^ https://github.com/bigdata-ustc/ekt

^9^ https://github.com/shshen-closer/Convolutional-Knowledge-Tracings

^10^ https://github.com/arghosh/AKT
